# Hopelessness in Police Officers and Its Association with Depression and Burnout: A Pilot Study

**DOI:** 10.3390/ijerph19095169

**Published:** 2022-04-24

**Authors:** Cristina Civilotti, Daniela Acquadro Maran, Sergio Garbarino, Nicola Magnavita

**Affiliations:** 1 Department of Psychology, Università di Torino, Via Verdi 10, 10124 Torino, Italy; cristina.civilotti@unito.it; 2 WOW-Work and Organizational Wellbeing Research Group, 10124 Torino, Italy; 3 Department of Neuroscience, Rehabilitation, Ophthalmology, Genetics and Maternal/Child Sciences (DI-14 NOGMI), University of Genoa, 16132 Genoa, Italy; sgarbarino.neuro@gmail.com; 4 Postgraduate School of Occupational Health, Università Cattolica del Sacro Cuore, 00168 Rome, Italy; nicola.magnavita@unicatt.it; 5 Department of Woman, Child & Public Health Sciences, Fondazione Policlinico Universitario A. Gemelli IRCCS, 00168 Rome, Italy

**Keywords:** suicidal ideation, mental health, depression, helping professions, stress

## Abstract

Hopelessness is a particularly critical condition and a risk factor for suicide. Many studies have reported that this condition is common in some occupations and is associated with high stress that is not properly managed. This study examined the prevalence of hopeless status (HS) in a sample of police officers (POs) and the association of hopelessness with depression, burnout, and suicidality. In total, 127 out of 231 POs participated in the survey; they were assessed with the Beck Hopelessness Scale, the Beck Depression Scale, and the Maslach Burnout Inventory. A total of 26.5% of POs reported hopelessness, and a significant association was found with depression and burnout; in individual cases, these conditions were associated with suicidal thoughts. In a multivariate logistic regression model adjusted for confounding variables, depression, emotional exhaustion, and reduction of personal accomplishment were significantly associated with HP status. Depression (OR = 3.02, 95% CI: 1–9.12) and emotional exhaustion (OR = 1.88, 95% CI: 1.06–3.32) significantly increased the risk of hopelessness, while personal accomplishment (OR = 0.57, 95% CI: 0.32–1) was a protective factor. Hopelessness appears to be a very important factor to consider when assessing POs’ mental health.

## 1. Introduction

First responders are often exposed to traumatic events [1,2,3,4,5,6] that can be stressful and affect mental health [7]. Police officers (POs) are highly exposed to chronic stressors such as dangerous situations, injuries, violence, and the need to report and testify about psychologically stressful scenes [8]. Consequently, the impact of these occupational activities on their mental and physical health is high [3,9]. If the stress is not properly managed, the negative consequences may include absenteeism [10], a decrease in psychological well-being [3], and a decreased ability to concentrate [11,12]. Chronic tension can also affect family relationships [13,14,15,16], physical health [9], and quality of life [17,18,19]. Mental health consequences of poorly managed occupational stress can include serious conditions, such as burnout, depression, and hopelessness.

### 1.1. Hopelessness, Depression, Burnout, and Suicide Risk in Police Officers

The concept of hopelessness was first proposed by Beck [20]. It is a cognitive/motivational state characterized by negative expectations about the future. More specifically, it represents the future-oriented component of Beck’s “cognitive triad model” [21] and indicates an information processing bias or schema that distorts the depressed person’s perception of external reality. From this perspective, depression and hopelessness are related but conceptually distinct [22,23,24,25]. For example, hopelessness plays an important role in mediating the relationship between depressive syndromes and suicidal behavior [26,27] and is therefore fundamental to suicide risk assessment and a good predictor of suicidality [28,29,30]. In distinguishing hopelessness from depression, many studies have also shown that the severity of suicidal intent appears to be more strongly related to hopelessness than depression and that hopelessness can lead to suicidal ideation or behavior [31,32,33,34,35,36,37,38]. The identification of hopeless individuals may be considered a “red flag” in helping professions such as nurses [39], physicians [40], teachers [41], and police officers [42] and should encourage preventive measures.

Burnout is a syndrome that results from chronic stress at work that has not been successfully managed [43]. It is traditionally described by the three dimensions of ex-exhaustion (feeling exhausted), cynicism and depersonalization (distant attitude toward colleagues and users), and decreased work effectiveness [44,45]. The factors that favor the occurrence of the phenomenon can be personal characteristics of the person, such as personality, or organizational characteristics related to the tasks to be performed and the work context [46,47,48]. Burnout is listed as an occupational phenomenon in the 11th revision of the International Classification of Diseases (ICD-11) [49] but is not classified as a medical condition. In POs, the effects of burnout can range from a mild degree of dysfunction to exhaustion [50]. According to Golembiewski and Kim [51], POs may suffer from a variety of burnout symptoms, including decreased self-esteem, lack of confidence in one’s work, decreased job satisfaction, relationship problems with colleagues and/or significant others, and difficulty keeping things in balance and making appropriate decisions. The physiological, psychological, and behavioral effects of stress and burnout in POs can be acute, which is of great concern. This is because hopelessness, burnout, and stress have been found to be interrelated [52], and policing is a profession characterized by very high levels of stress [53,54,55,56] and, according to some studies, higher suicidality compared to the general population [57,58,59,60]. As stated by the American Psychological Association, the term “suicidality” refers to “the risk of suicide, usually indicated by suicidal thoughts or intentions, especially when a well-developed suicide plan is in place” [61].

This study focuses on the concept of hopelessness as a key factor in understanding police officers’ experiences in the face of overwhelming stress. Studying hopelessness and its association with burnout, depression, and suicidal ideation could be an important issue in preventing suicidality in POs.

### 1.2. Objective of the Research

Although much research has examined aspects of depression, mainly as a general clinical or subclinical condition, and burnout in POs, few studies have focused on hopelessness, which is considered a key factor in preventing suicidal and parasuicidal acts [42]. The present study seeks to address this gap by examining the prevalence of hopelessness in a sample of POs. We also hypothesize:

1. A correlation of hopelessness with sociodemographic variables;
2. A correlation with depression, suicidal ideation, and the three dimensions of burnout (emotional exhaustion, depersonalization, and personal fulfillment).

Finally, we tested the relationship between hopelessness and mental health indicators (depression, burnout components) using a multivariate logistic regression model.

## 2. Materials and Methods

### 2.1. Ethics and Procedure

This research complies with the provisions of the Declaration of Helsinki [62], and all ethical guidelines required for the conduct of research involving human subjects were followed, including compliance with Italian legal requirements for the study and the rules of the Code of Ethics of the Order of Italian Psychologists. After approval by the Ethics Committee of the University of Turin (protocol number: 14526), the leaders of three commands in three small towns (using the definition of town size proposed by the Italian National Institute of Statistics [63]) were contacted. After the command gave its consent, an internal communication announced the participation of the University of Turin in the study. An email was sent to the institutional addresses of the POs to point out the objectives of the research and the ethical aspects. Subsequently, the questionnaires and interviews were completed. The administration period was between November 2019 and September 2021.

The sample consisted of 127 POs from 231 POs. The POs contacted for the study were selected using a stratified random procedure, with different command units, gender, and role as the basis for stratification. In total, 231 POs were selected, and 127 of them (55.2%) accepted to participate in the survey and signed the informed consent form.

Participants were assured of the anonymity of their responses by following ethical guidelines for conducting interviews. Participation in the survey was voluntary, and participants received no compensation for their participation. The estimated time required to answer the questions was approximately 30–40 min.

### 2.2. Materials

Participants were given a questionnaire that consisted of the following scales in addition to a module with sociodemographic characteristics:

BHS. The Beck Hopelessness Scale [64,65] is a scale designed to measure the cognitive component of depression, focusing on three important aspects: feelings about the future, loss of motivation, and expectations. The scale consists of 20 items (9 statements are classified as false and 11 as true), and subjects can choose to agree or disagree with the statement. A score of 0 or 1 is assigned for each statement, and the total hopelessness score is the sum of the 20 items. Higher scores indicate higher levels of hopelessness. The literature supports the positive correlation between BHS scores and measures of depression and suicidal thoughts and intentions. Beck et al. [31] observed that some psychiatric patients who had committed suicide had reported a score of 9 or higher. For this reason, the BHS score can be used as an indicator of suicidality. We classified individuals with a score of 9 or more on the BHS scale as “hopeless person” (HP). The BHS scale showed good internal consistency in nonclinical samples [66] and has also been used in police [42,67]. The reliability coefficient in this sample was 0.81.

BDI- 13. The Beck Depression Inventory [68,69] is a self-report instrument for depression. In its full version, it consists of 21 items, each of which refers to a specific category of symptoms. The short form of the BDI, the BDI-13, was used in this study [70]. The thirteen items of the short form were selected because they had the highest correlations with the BDI total score and clinician ratings. Correlations with the 21-item version were 94 or higher. These results suggest that the short form is an acceptable substitute for the long form [21]. Each item consists of 4 sentences indicating the presence or absence of negative thoughts or feelings. Each of these sentences is assigned a value between 0 and 3. As indicated by Dadfar and Kalibatseva [68], the following cut-off norms were used: normal (0–3); mild depression (4–7); mild to moderate depression (8–11); moderate depression (12–15); and severe depression (16–39). The reliability coefficient for the population of this study was 0.86.

One of the items of the BDI relates to suicidal thoughts (statements ranging from “I have no thoughts of killing myself” = 0 to “I would kill myself if I had the opportunity” = 3) and has been used to assess suicidal thoughts. Green et al. [69] have shown that a high BDI suicide item score significantly predicts both deaths by suicide and repeated suicide attempts in psychiatric patients. In this study, we considered scores ≥1 in this item as an indicator of suicidal thoughts.

MBI. The Maslach Burnout Inventory [70,71] is a burnout assessment instrument consisting of three subscales: emotional exhaustion (MBI_EE), depersonalization (MBI_D), and personal accomplishment (MBI_PA). The scale consists of a total of 22 items with a response option ranging from 0 (never) to 6 (every day). The total score is calculated by adding the individual responses to each item; cut-off values are usually calculated for each subscale. The reliability coefficient for the population of the present study was 0.77.

### 2.3. Statistical Analysis

Statistical analyses performed included: descriptive frequency analyses for categorical variables with calculation of percentage and valid percentage; descriptive analyses of mean and standard deviation for numerical variables; chi-square analyses for distribution between groups in relation to categorical variables; and Pearson’s r statistic for evaluation of correlations for continuous variables.

The relationship between mental health indicators (depression, components of burnout) and HP status was examined using a multivariate logistic regression model with HP status as the dependent variable) and depression (BDI score), emotional exhaustion, depersonalization, and personal coping as predictors. In this way, odds ratio (OR) and 95% confidence interval (CI95%) for the occurrence of HP status were calculated.

The statistical analysis software IBM/SPSS, ver. 27.0 (IBM SPSS Statistics for Macintosh, IBM Corp., Armonk, NY, USA) [72] was used for data analysis.

### 2.4. Sample

The sample consisted of 127 subjects operating in an area in northwestern Italy. The average age of the subjects in the sample was 49.86 years (range 27–65, SD = 8.54), while the average working age was 20.09 years (range 1–41, SD = 10.13). Table 1 shows the other sociodemographic and work-related descriptive variables of the sample.

## 3. Results

There were no differences in BHS scores in relation to sociodemographic variables (gender, age, working age, being in a stable relationship, children) and in relation to work variables (work shift, task, sector) (data not shown). Thirty-one subjects (26.5%) had a BHS score above the cut-off (HP status), indicating a potentially critical situation.

BDI score indicated that nine subjects (7.6%) had mild depression, six subjects (5.1%) had moderate depression, and four (3.4%) had major depression. With reference to the suicide item of the BDI, five subjects (4.1%) reported a value ≥1 (three subjects selected the response “I have thoughts of killing myself, but I would not carry them out”, one subject selected the response “I would like to kill myself”, and finally, one subject selected the response “I would kill myself if I had the opportunity”). HP status was present in four out of five subjects who reported suicidal thoughts.

Table 2 shows the minimum and maximum values, means, and standard deviations of BDI, BSH, and the three subscales of MBI. Using the cut-off values for the MBI_EE subscale, 24 subjects (18.9%) reported a high MBI_EE level, 34 (26.8%) a medium MBI_EE level, and 69 (54.3%) a low level. For the BMI_D subscale, 34 subjects (26.8%) reported a high MBI_D level, 22 (17.3%) reported a medium MBI_EE level, and 71 (55.9%) reported a low MBI_D level. Finally, for the BMI_PA subscale, which is usually inversely related to the other two dimensions, 69 subjects (54.3%) indicated a low level of MBI_PA, 42 (33.1%) indicated a medium level of MBI_PA, and 34 subjects (12.6%) indicated a high level of MBI_PA.

Table 3 shows the correlations between hopelessness and the variables studied. Age and working age were not correlated with BHS, whereas the BDI total score and the BDI item on suicide, as well as the three burnout variables, were positively correlated with BHS.

A multivariate logistic regression model was used to assess the relationship between depression, burnout, and hopelessness. After adjustment for sociodemographic and work-related variables, depression, emotional exhaustion, and reduction of personal accomplishment were significantly associated with HP status. The logistic regression model was statistically significant, χ^2^(17) = 97.361, *p* < 0.001, and explained 89% (Nagelkerke R^2^) of the variance in hopelessness (Table 4).

## 4. Discussion

In this pilot study, a significant percentage of POs were found to be in a state of hopelessness. Depression and burnout were significant predictors of hopelessness. Some of the hopeless POs had suicidal thoughts.

The scientific literature has already shown that the consequences of hopelessness, burnout, negative emotions, and future expectations can be severe [73,74,75]. This is especially true for a population exposed to work-related stressors, such as POs. The consequences of a chronic state of distress can affect a variety of domains related to both the individual sphere (emotional, relational, and cognitive) and productivity via the spillover effect [14], such as higher absenteeism, greater difficulty concentrating, poorer interaction with users and colleagues, lower work motivation, greater disengagement, and in extreme cases, suicidal thoughts or behaviors [42,76,77]. For these reasons, it is important to better understand the phenomenon to create targeted and effective intervention options.

As a first result, it is noteworthy that in our sample, there was a high prevalence of hopelessness (26.5%). There is insufficient evidence in the literature to indicate whether this percentage is common among POs or in other workplaces. Conversely, we can compare the rate of depression observed in our sample (16.1%) with that derived from a me-ta analysis of mental health in the police [78], which estimated an overall pooled prevalence of 14.6%, with a confidence interval (95% CI: from 10.9% to 18.6%) that includes the values we observed. We can therefore assume that the observed situation in which more than one in four POs is hopeless could also occur in other police forces.

Hopelessness is particularly important from a clinical perspective because of several factors. Especially in terms of prevention, as hopelessness was found to be 1.3 times more important than depression in explaining suicidal ideation [79]. Moreover, this finding provides a remarkable clinical indication of how to attend to the psychophysiological well-being of POs by addressing specific interventions. In 2016, Violanti et al. [42] examined the associations between hopelessness and work-related stress, stratified by PTSD symptoms, in a population of POs. They found that work-related stress was associated with an increase in hopelessness. This finding may suggest that stress, especially when associated with PTSD symptoms, creates a dimension of perceived loss of control and a sense of the immutability of the external context [80]. Another cause of hopelessness lies in the negative aspects and perceived ineffectiveness of their work [81]. For example, POs may have difficulty connecting with users, recently especially through social media [82,83], or more generally feel that their crime-fighting efforts are in vain [84]. In addition, many studies reported that a combination of high workload and low autonomy, especially in the decision-making phase, is likely to lead to job dissatisfaction and health problems and may result in overwhelming feelings of hopelessness [85,86,87,88].

Disconfirming the first hypothesis, no differences in the level of hopelessness were found in this study in relation to sociodemographic variables (gender, age, steady relationship, and children) or in relation to work variables (work shifts, tasks, and sections). This homogeneous distribution of hopelessness across gender, age groups, and the different work parameters studied have already been noted in the literature [89,90]. There are other risk factors that influence the development of hopelessness. For example, Violanti et al. [42] suggest that administrative practices and lack of departmental support have a greater impact on employees’ perceptions of hopelessness than the risk of working on the street.

In support of the second hypothesis, significant correlations were found between hopelessness, burnout, and depression. It appears that those POs who are more likely to exhibit depressive traits and suffer from burnout are also more likely to be hopeless. From this perspective, a lack of control over one’s work, disappointed hopes and expectations, and a sense of losing the meaning of life seem to be related to burnout. Both are associated with a general feeling of emptiness, which may be accompanied by a decline in self-esteem [91,92,93,94].

Consistent with previous research showing that hopelessness is often a critical element in suicidal thoughts and attempts [95], hopelessness was present in four out of five subjects who reported suicidal thoughts. In addition to factors such as lack of organizational support and PTSD, other work-related risk factors for suicide in POs that may be related to the construct of hopelessness are also reported in the literature. For example, problems related to domestic relationships [96,97,98], abuse of alcohol and/or other substances [88,99], and easy access to weapons even in moments of emotional fragility are very frequently cited as precursors to suicide or correlates of suicidal ideation [100]. In this context, it is important to point out that many POs are not adequately trained about suicide [101,102,103,104] and that there is the long-standing problem of social stigmatization of police officers in case of perceived danger [105,106]: this can increase the level of hopelessness about the future and lead to a vicious circle characterized by a kind of “fear without solution” [107]. The prevalence of suicidal ideation (4.1%) in our sample was below the limits observed in the above-cited meta-analysis on mental health in police, in which the estimated point prevalence of suicidal ideation was 8.5% (95% CI 6.1% to 11.2%) [78]. Nonetheless, it should be noted that some POs in our sample have reported suicidal thoughts, which may be a call for help. As a result, we have provided the administration with our availability for psychological support interventions for the OPs in need. It should be remembered that the POs are provided with firearms, which increases the risk of suicide [108]. On the other hand, it is known that POs find considerable barriers to resorting to psychiatric treatments [109] due to the fear of negative repercussions on their career [110,111].

The results of this study suggest that burnout and depression in POs need to be addressed as preventive factors to reduce hopelessness in this population. In the case of hopelessness, especially when it is accompanied by burnout and/or depression, it is important to increase POs’ self-confidence and self-efficacy regarding work-related issues. In addition to educational support to reduce stress, which can cause physical and mental suffering, specialized psychological support is essential to reduce hopelessness.

Although this study has the distinct advantage of directly examining the relationship between hopelessness and suicidal thoughts and provides an important clinical framework for understanding the construct of hopelessness in POs, it has several limitations. First, because of the cross-sectional design and correlational nature of the data, causal relationships between burnout syndromes, depression, and hopelessness could not be established even if they were plausible, and these findings warrant further investigation. Longitudinal studies could examine whether there are influential mediators of the association between burnout, depression, and hopelessness. Second, participant self-selection may have introduced selection bias. Third, as with all self-completed questionnaires, the lack of objective measures means that it cannot be ruled out that social desirability may have led to response bias [112]. Fourth, the COVID-19 pandemic may have been a risk factor that increased feelings of hopelessness [113,114].

## 5. Conclusions

The present study showed that hopelessness is serious and widespread among police officers. From the results of this study, we concluded that hopelessness among police officers can be explained by their level of depression, emotional exhaustion, and personal performance. These findings have an important policy or practical implications. We hypothesized that hopelessness is a manifestation of psychological vulnerability and, along with burnout and depression, may increase the risk of suicidality.

Hopelessness as one of the potential negative mental health outcomes for police officers must be prevented and treated at all costs. It is important to implement programs and interventions that target specific workplace health problems, especially those related to hopelessness, emotional exhaustion, and depression. To improve intrinsic motivation and quality of life, it is also necessary to implement training interventions that provide useful psychological tools and skills for coping with the many and varied forms of stress. This type of intervention is also appropriate to increase prophylactic factors for suicide risk, which has been shown to be significantly associated with emotional exhaustion in the workplace. Police organizations may consider further developing organizational structural factors to improve stress management support and reduce burnout risk. These factors are adaptable and can be implemented in very different and competent ways. Future studies should examine this area with larger samples and could consider other risk factors such as socioeconomic variables, ethnicity, and other organizational and occupational factors. Furthermore, additional studies in other police departments with different characteristics are essential to generalize these findings.

## Figures and Tables

**Table 1:** Sociodemographic and work-related characteristics of the sample.

Descriptive Variables	N	Valid %
***Sociodemographic characteristics*** **Gender**		
Male	70	56
Female	55	44
**Relational status**		
Stable relationship	98	80.3
No relationship	24	19.7
**Marital status**		
Unmarried	25	20.2
Married	78	62.9
Separated	7	5.6
Divorced	9	7.3
Widowed	5	4.0
**Sons**		
Yes	88	71.5
No	35	28.5
***Work-related characteristics*** **Section**		
Surveillance	106	87.6
Administration	15	12.4
**Shift**		
Day shift	92	75.4
24-h shift	30	24.6
**Role**		
Agents	98	79.7
Chiefs	25	20.3

**Table 2:** Mean scores for hopelessness, depression, and burnout scales.

Variables	Min	Max	Mean	SD
Depression (BDI)	0	19	4.02	4.34
Hopelessness (BHS)	0	18	5.45	4.33
Emotional exhaustion (MBI_EE)	2	47	17.80	9.77
Depersonalization (MBI_D)	0	27	6.33	6.15
Personal accomplishments (MBI_PA)	11	44	31.24	7.39

**Table 3:** Correlations of hopelessness with age, working age, depression, suicidal thoughts, and burnout subscales.

Variables	BHS
Age	−0.12
Working age	−0.01
Depression (BDI)	0.64 **
Suicidal thoughts (BDI item on suicide)	0.39 **
Emotional exhaustion (MBI_EE)	0.49 **
Depersonalization (MBI_D)	0.38 **
Personal accomplishment (MBI_PA)	−0.33 **

Note: * *p <* 0.05; ** *p* < 0.01; *** *p* < 0.001.

**Table 4:** Association of depression and burnout with hopeless status. Adjusted odds ratios and confidence intervals.

	*p*	Adjusted OR	95% CI	
Depression (BDI)	0.05	3.02	1.00	9.12
Emotional exhaustion (MBI_EE)	0.03	1.88	1.06	3.32
Depersonalization (MBI_D)	0.20	0.73	0.45	1.18
Personal accomplishment (MBI_PA)	0.05	0.57	0.32	1.00

Note: Adjusted for sociodemographic variables (age, gender, relational and marital status, presence of children) and work-related characteristics (occupational section, work shift, role). CI—confidence Interval.

## Data Availability

The data presented in this study are available on request from the corresponding author. The data are not publicly available due the consent provided by participants on the use of confidential data.
